# Hexane-1,6-diaminium bis­[3,4,5,6-tetra­chloro-2-(meth­oxy­carbon­yl)benzoate]

**DOI:** 10.1107/S1600536811008506

**Published:** 2011-03-15

**Authors:** Jian Li

**Affiliations:** a Department of Chemistry and Chemical Engineering, Weifang University, Weifang 261061, People’s Republic of China

## Abstract

In the anion of the title salt, C_6_H_18_N_2_^2+^·2C_9_H_3_Cl_4_O_4_^−^, the meth­oxy­carbonyl and carboxyl groups are aligned at dihedral angles of 71.0 (3) and 100.9 (3)°, respectively, with the aromatic ring. The asymmetric unit contains half a cation and one anion. In the crystal, inter­molecular N—H⋯O, C—H⋯Cl and C—H⋯O hydrogen bonds link the components into a three-dimensional network.

## Related literature

For related structures, see: Li (2011 ▶); Liang (2008 ▶).
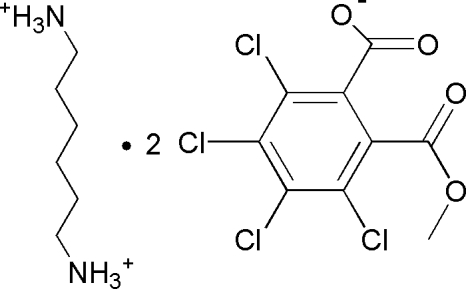


## Experimental

### 

#### Crystal data

C_6_H_18_N_2_^2+^·2C_9_H_3_Cl_4_O_4_^−^
*M* *_r_* = 752.05
Monoclinic, 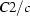

*a* = 31.236 (3) Å
*b* = 5.8911 (4) Å
*c* = 18.3762 (18) Å
β = 107.118 (1)°
*V* = 3231.7 (5) Å^3^
*Z* = 4
Mo *K*α radiation
μ = 0.74 mm^−1^
*T* = 298 K
0.37 × 0.28 × 0.15 mm

#### Data collection

Bruker SMART CCD diffractometer
Absorption correction: multi-scan (*SADABS*; Bruker, 1997 ▶) *T*_min_ = 0.770, *T*_max_ = 0.897
7618 measured reflections
2829 independent reflections
1817 reflections with *I* > 2σ(*I*)
*R*_int_ = 0.037

#### Refinement

*R*[*F* ^2^ > 2σ(*F* ^2^)] = 0.057
*wR*(*F* ^2^) = 0.154
*S* = 1.04
2829 reflections
192 parameters
H-atom parameters constrained
Δρ_max_ = 0.51 e Å^−3^
Δρ_min_ = −0.41 e Å^−3^

### 

Data collection: *SMART* (Bruker, 1997 ▶); cell refinement: *SAINT* (Bruker, 1997 ▶); data reduction: *SAINT*; program(s) used to solve structure: *SHELXS97* (Sheldrick, 2008 ▶); program(s) used to refine structure: *SHELXL97* (Sheldrick, 2008 ▶); molecular graphics: *SHELXTL* (Sheldrick, 2008 ▶); software used to prepare material for publication: *SHELXTL*.

## Supplementary Material

Crystal structure: contains datablocks global, I. DOI: 10.1107/S1600536811008506/bt5475sup1.cif

Structure factors: contains datablocks I. DOI: 10.1107/S1600536811008506/bt5475Isup2.hkl

Additional supplementary materials:  crystallographic information; 3D view; checkCIF report

## Figures and Tables

**Table 1:** Hydrogen-bond geometry (Å, °)

*D*—H⋯*A*	*D*—H	H⋯*A*	*D*⋯*A*	*D*—H⋯*A*
N1—H1*A*⋯O4	0.89	1.90	2.770 (5)	165
N1—H1*B*⋯O3^i^	0.89	1.87	2.757 (5)	171
C9—H9*B*⋯Cl4^ii^	0.96	2.75	3.677 (9)	161
C10—H10*B*⋯O2	0.97	2.58	3.208 (7)	122

Symmetry codes: (i) 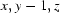; (ii) 
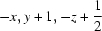
.

**Fig. 1.**
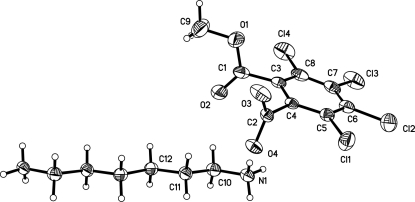
The molecular structure of (I), drawn with 30% probability ellipsoids.

**Fig. 2.**
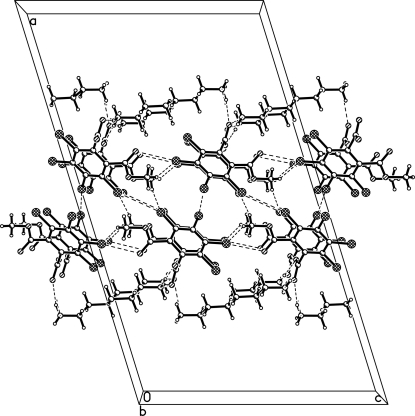
The crystal packing of (I), viewed along b axis. Hydrogen bonds are indicated by dashed lines.

## supplementary crystallographic information

### Comment

In the present work, the reaction of 3,4,5,6-tetrachloro-2-(methoxycarbonyl)benzoic acid and hexane-1,6-diamine in methanol is expected to yield 4,5,6,7-tetrachloro-2-[6-(4,5,6,7- tetrachloro-1,3-dioxoisoindolin-2-yl)hexyl]isoindoline-1,3-dione. However, the product is hexane-1,6-diaminium 3,4,5,6-tetrachloro-2-(methoxycarbonyl)benzoate (Scheme I, Fig. 1), this may be the reason of a shorter time and cooler temperature in the reaction. The asymmetric unit of the title compound (I) contains half a hexane-1,6-diaminium cation and one 3,4,5,6-tetrachloro-2-(methoxycarbonyl)benzoate anion (Fig. 1). In the anion of the title salt, the methoxycarbonyl and carboxyl groups are aligned at dihedral angles of 71.0 (3) and 100.9 (3) °, respectively, with the aromatic ring. The bond lengths and angles are in agreement with those in ethylammonium 2-(methoxycarbonyl)-3,4,5,6-tetrabromobenzoate methanol solvate (Li, 2011) and in ethane-1,2-diammonium bis(2-(methoxycarbonyl)-3,4,5,6-tetrabromobenzoate) methanol solvate (Liang, 2008). In the crystal structure, intermolecular N—H···O, C—H···Cl and C—H···O hydrogen bonds link the components of the structure into three-dimensional network (Fig. 2 and Table 1).

### Experimental

A mixture of 4,5,6,7-tetrachloroisobenzofuran-1,3-dione (2.86 g, 0.01 mol) and methanol (15 ml) was refluxed for 0.5 h. And then hexane-1,6-diamine (0.58 g, 0.005 mol) was added to the above solution, being mixed round for 20 min at room temperature. And then the solution was kept at room temperature for 6 d. Natural evaporation gave colourless single crystals of the title compound, suitable for X-ray analysis.

### Refinement

H atoms were initially located from difference maps and then refined in a riding model with C—H = 0.96–0.97 Å, N—H = 0.89 Å,   and *U*_iso_(H) = 1.2*U*_eq_(C) or 1.5*U*_eq_(O, N, methyl C).

### Crystal data

**Table:**

C_6_H_18_N_2_^2+^·2C_9_H_3_Cl_4_O_4_^−^	*F*(000) = 1528
*M**_r_* = 752.05	*D*_x_ = 1.546 Mg m^−^^3^
Monoclinic, *C*2/*c*	Mo *K*α radiation, λ = 0.71073 Å
*a* = 31.236 (3) Å	Cell parameters from 2053 reflections
*b* = 5.8911 (4) Å	θ = 2.7–26.1°
*c* = 18.3762 (18) Å	µ = 0.74 mm^−^^1^
β = 107.118 (1)°	*T* = 298 K
*V* = 3231.7 (5) Å^3^	Block, colorless
*Z* = 4	0.37 × 0.28 × 0.15 mm

### Data collection

**Table:**

Bruker SMART CCD diffractometer	2829 independent reflections
Radiation source: fine-focus sealed tube	1817 reflections with *I* > 2σ(*I*)
graphite	*R*_int_ = 0.037
φ and ω scans	θ_max_ = 25.0°, θ_min_ = 2.3°
Absorption correction: multi-scan (*SADABS*; Bruker, 1997)	*h* = −36→29
*T*_min_ = 0.770, *T*_max_ = 0.897	*k* = −6→7
7618 measured reflections	*l* = −21→21

### Refinement

**Table:**

Refinement on *F*^2^	Primary atom site location: structure-invariant direct methods
Least-squares matrix: full	Secondary atom site location: difference Fourier map
*R*[*F*^2^ > 2σ(*F*^2^)] = 0.057	Hydrogen site location: inferred from neighbouring sites
*wR*(*F*^2^) = 0.154	H-atom parameters constrained
*S* = 1.03	*w* = 1/[σ^2^(*F*_o_^2^) + (0.0489*P*)^2^ + 12.9951*P*] where *P* = (*F*_o_^2^ + 2*F*_c_^2^)/3
2829 reflections	(Δ/σ)_max_ < 0.001
192 parameters	Δρ_max_ = 0.51 e Å^−^^3^
0 restraints	Δρ_min_ = −0.41 e Å^−^^3^

### Special details

**Table:**

Geometry. All e.s.d.'s (except the e.s.d. in the dihedral angle between two l.s. planes) are estimated using the full covariance matrix. The cell e.s.d.'s are taken into account individually in the estimation of e.s.d.'s in distances, angles and torsion angles; correlations between e.s.d.'s in cell parameters are only used when they are defined by crystal symmetry. An approximate (isotropic) treatment of cell e.s.d.'s is used for estimating e.s.d.'s involving l.s. planes.
Refinement. Refinement of *F*^2^ against ALL reflections. The weighted *R*-factor *wR* and goodness of fit *S* are based on *F*^2^, conventional *R*-factors *R* are based on *F*, with *F* set to zero for negative *F*^2^. The threshold expression of *F*^2^ > σ(*F*^2^) is used only for calculating *R*-factors(gt) *etc*. and is not relevant to the choice of reflections for refinement. *R*-factors based on *F*^2^ are statistically about twice as large as those based on *F*, and *R*- factors based on ALL data will be even larger.

### Fractional atomic coordinates and isotropic or equivalent isotropic displacement parameters (Å^2^)

**Table:**

	*x*	*y*	*z*	*U*_iso_*/*U*_eq_	
Cl1	0.16469 (4)	0.8320 (3)	0.02548 (7)	0.0738 (5)	
Cl2	0.10240 (5)	0.4349 (3)	−0.04960 (8)	0.0845 (5)	
Cl3	0.03378 (5)	0.2434 (2)	0.02771 (10)	0.0912 (6)	
Cl4	0.02472 (5)	0.4714 (3)	0.17526 (10)	0.0918 (6)	
N1	0.21033 (11)	0.4754 (6)	0.26915 (19)	0.0485 (9)	
H1A	0.2012	0.6131	0.2512	0.073*	
H1B	0.1918	0.3715	0.2417	0.073*	
H1C	0.2379	0.4510	0.2662	0.073*	
O1	0.06089 (14)	0.9497 (7)	0.2473 (2)	0.0906 (13)	
O2	0.12386 (14)	0.7821 (10)	0.3033 (2)	0.1124 (18)	
O3	0.14859 (11)	1.1892 (6)	0.1748 (2)	0.0769 (12)	
O4	0.19786 (10)	0.9160 (5)	0.21430 (17)	0.0537 (8)	
C1	0.09506 (15)	0.8248 (9)	0.2481 (3)	0.0558 (12)	
C2	0.16017 (13)	0.9909 (7)	0.1788 (2)	0.0400 (10)	
C3	0.09403 (13)	0.7360 (7)	0.1712 (2)	0.0432 (10)	
C4	0.12605 (12)	0.8139 (6)	0.1380 (2)	0.0373 (9)	
C5	0.12705 (13)	0.7243 (7)	0.0688 (2)	0.0458 (10)	
C6	0.09835 (14)	0.5502 (7)	0.0339 (3)	0.0511 (12)	
C7	0.06762 (15)	0.4685 (8)	0.0678 (3)	0.0559 (13)	
C8	0.06449 (14)	0.5651 (8)	0.1346 (3)	0.0542 (12)	
C9	0.0599 (3)	1.0419 (13)	0.3203 (3)	0.122 (3)	
H9A	0.0605	0.9197	0.3552	0.184*	
H9B	0.0330	1.1290	0.3134	0.184*	
H9C	0.0855	1.1377	0.3405	0.184*	
C10	0.21042 (15)	0.4596 (8)	0.3498 (3)	0.0531 (12)	
H10A	0.2253	0.3210	0.3722	0.064*	
H10B	0.1798	0.4547	0.3523	0.064*	
C11	0.23434 (15)	0.6622 (8)	0.3940 (2)	0.0498 (11)	
H11A	0.2645	0.6693	0.3894	0.060*	
H11B	0.2188	0.7997	0.3718	0.060*	
C12	0.23681 (14)	0.6522 (8)	0.4771 (2)	0.0517 (11)	
H12A	0.2507	0.5102	0.4983	0.062*	
H12B	0.2066	0.6541	0.4816	0.062*	

### Atomic displacement parameters (Å^2^)

**Table:**

	*U*^11^	*U*^22^	*U*^33^	*U*^12^	*U*^13^	*U*^23^
Cl1	0.0691 (9)	0.0891 (11)	0.0710 (8)	−0.0250 (7)	0.0329 (7)	−0.0234 (7)
Cl2	0.0825 (10)	0.0811 (11)	0.0783 (9)	−0.0055 (8)	0.0057 (8)	−0.0407 (8)
Cl3	0.0688 (9)	0.0500 (8)	0.1278 (13)	−0.0243 (7)	−0.0131 (9)	−0.0073 (8)
Cl4	0.0664 (9)	0.0909 (12)	0.1210 (13)	−0.0240 (8)	0.0322 (9)	0.0288 (10)
N1	0.044 (2)	0.033 (2)	0.060 (2)	0.0003 (16)	0.0021 (17)	−0.0084 (17)
O1	0.112 (3)	0.090 (3)	0.067 (2)	0.053 (3)	0.021 (2)	0.007 (2)
O2	0.094 (3)	0.181 (5)	0.058 (2)	0.071 (3)	0.016 (2)	0.010 (3)
O3	0.063 (2)	0.035 (2)	0.105 (3)	0.0031 (16)	−0.019 (2)	−0.0100 (18)
O4	0.0404 (17)	0.0431 (18)	0.0658 (19)	−0.0031 (14)	−0.0025 (15)	0.0026 (15)
C1	0.041 (3)	0.066 (3)	0.061 (3)	0.012 (2)	0.015 (2)	0.018 (3)
C2	0.038 (2)	0.035 (3)	0.043 (2)	−0.0057 (19)	0.0049 (18)	−0.0034 (19)
C3	0.034 (2)	0.037 (2)	0.054 (3)	0.0065 (19)	0.0067 (19)	0.010 (2)
C4	0.031 (2)	0.027 (2)	0.047 (2)	0.0021 (16)	0.0018 (17)	−0.0004 (18)
C5	0.037 (2)	0.041 (3)	0.055 (3)	−0.0035 (19)	0.007 (2)	−0.008 (2)
C6	0.041 (2)	0.036 (3)	0.065 (3)	0.002 (2)	−0.002 (2)	−0.011 (2)
C7	0.040 (3)	0.036 (3)	0.075 (3)	−0.004 (2)	−0.008 (2)	−0.003 (2)
C8	0.035 (2)	0.045 (3)	0.076 (3)	−0.003 (2)	0.007 (2)	0.019 (3)
C9	0.171 (7)	0.127 (6)	0.072 (4)	0.084 (6)	0.042 (4)	0.010 (4)
C10	0.048 (3)	0.046 (3)	0.063 (3)	−0.001 (2)	0.012 (2)	−0.003 (2)
C11	0.049 (3)	0.042 (3)	0.053 (3)	0.002 (2)	0.007 (2)	−0.007 (2)
C12	0.045 (3)	0.052 (3)	0.057 (3)	0.001 (2)	0.014 (2)	−0.007 (2)

### Geometric parameters (Å, °)

**Table:**

Cl1—C5	1.723 (5)	C4—C5	1.386 (6)
Cl2—C6	1.716 (5)	C5—C6	1.389 (6)
Cl3—C7	1.721 (4)	C6—C7	1.376 (7)
Cl4—C8	1.719 (5)	C7—C8	1.382 (7)
N1—C10	1.484 (5)	C9—H9A	0.9600
N1—H1A	0.8900	C9—H9B	0.9600
N1—H1B	0.8900	C9—H9C	0.9600
N1—H1C	0.8900	C10—C11	1.511 (6)
O1—C1	1.293 (5)	C10—H10A	0.9700
O1—C9	1.456 (7)	C10—H10B	0.9700
O2—C1	1.168 (5)	C11—C12	1.507 (6)
O3—C2	1.218 (5)	C11—H11A	0.9700
O4—C2	1.247 (5)	C11—H11B	0.9700
C1—C3	1.499 (6)	C12—C12^i^	1.519 (9)
C2—C4	1.521 (5)	C12—H12A	0.9700
C3—C4	1.394 (6)	C12—H12B	0.9700
C3—C8	1.397 (6)		
			
C10—N1—H1A	109.5	C7—C8—C3	121.0 (4)
C10—N1—H1B	109.5	C7—C8—Cl4	120.2 (4)
H1A—N1—H1B	109.5	C3—C8—Cl4	118.8 (4)
C10—N1—H1C	109.5	O1—C9—H9A	109.5
H1A—N1—H1C	109.5	O1—C9—H9B	109.5
H1B—N1—H1C	109.5	H9A—C9—H9B	109.5
C1—O1—C9	116.3 (4)	O1—C9—H9C	109.5
O2—C1—O1	123.7 (5)	H9A—C9—H9C	109.5
O2—C1—C3	122.7 (4)	H9B—C9—H9C	109.5
O1—C1—C3	113.6 (4)	N1—C10—C11	110.0 (4)
O3—C2—O4	126.1 (4)	N1—C10—H10A	109.7
O3—C2—C4	118.3 (4)	C11—C10—H10A	109.7
O4—C2—C4	115.6 (4)	N1—C10—H10B	109.7
C4—C3—C8	119.0 (4)	C11—C10—H10B	109.7
C4—C3—C1	118.6 (4)	H10A—C10—H10B	108.2
C8—C3—C1	122.2 (4)	C12—C11—C10	112.5 (4)
C5—C4—C3	119.3 (4)	C12—C11—H11A	109.1
C5—C4—C2	120.8 (4)	C10—C11—H11A	109.1
C3—C4—C2	120.0 (4)	C12—C11—H11B	109.1
C4—C5—C6	121.3 (4)	C10—C11—H11B	109.1
C4—C5—Cl1	119.1 (3)	H11A—C11—H11B	107.8
C6—C5—Cl1	119.6 (3)	C11—C12—C12^i^	113.0 (5)
C7—C6—C5	119.4 (4)	C11—C12—H12A	109.0
C7—C6—Cl2	120.7 (3)	C12^i^—C12—H12A	109.0
C5—C6—Cl2	119.9 (4)	C11—C12—H12B	109.0
C6—C7—C8	120.0 (4)	C12^i^—C12—H12B	109.0
C6—C7—Cl3	119.9 (4)	H12A—C12—H12B	107.8
C8—C7—Cl3	120.1 (4)		
			
C9—O1—C1—O2	1.0 (9)	C4—C5—C6—C7	1.5 (6)
C9—O1—C1—C3	−179.9 (5)	Cl1—C5—C6—C7	−178.4 (3)
O2—C1—C3—C4	−68.0 (7)	C4—C5—C6—Cl2	−177.0 (3)
O1—C1—C3—C4	112.9 (5)	Cl1—C5—C6—Cl2	3.1 (5)
O2—C1—C3—C8	106.7 (6)	C5—C6—C7—C8	2.3 (6)
O1—C1—C3—C8	−72.4 (6)	Cl2—C6—C7—C8	−179.3 (3)
C8—C3—C4—C5	1.6 (6)	C5—C6—C7—Cl3	−176.7 (3)
C1—C3—C4—C5	176.5 (4)	Cl2—C6—C7—Cl3	1.7 (5)
C8—C3—C4—C2	−176.8 (4)	C6—C7—C8—C3	−4.1 (7)
C1—C3—C4—C2	−1.8 (5)	Cl3—C7—C8—C3	174.9 (3)
O3—C2—C4—C5	101.7 (5)	C6—C7—C8—Cl4	177.5 (3)
O4—C2—C4—C5	−79.0 (5)	Cl3—C7—C8—Cl4	−3.6 (5)
O3—C2—C4—C3	−79.9 (5)	C4—C3—C8—C7	2.1 (6)
O4—C2—C4—C3	99.4 (4)	C1—C3—C8—C7	−172.6 (4)
C3—C4—C5—C6	−3.4 (6)	C4—C3—C8—Cl4	−179.4 (3)
C2—C4—C5—C6	174.9 (4)	C1—C3—C8—Cl4	5.9 (6)
C3—C4—C5—Cl1	176.5 (3)	N1—C10—C11—C12	−178.2 (4)
C2—C4—C5—Cl1	−5.2 (5)	C10—C11—C12—C12^i^	176.6 (5)

Symmetry codes: (i) −*x*+1/2, −*y*+3/2, −*z*+1.

### Hydrogen-bond geometry (Å, °)

**Table:**

*D*—H···*A*	*D*—H	H···*A*	*D*···*A*	*D*—H···*A*
N1—H1A···O4	0.89	1.90	2.770 (5)	165
N1—H1B···O3^ii^	0.89	1.87	2.757 (5)	171
C9—H9B···Cl4^iii^	0.96	2.75	3.677 (9)	161
C10—H10B···O2	0.97	2.58	3.208 (7)	122

Symmetry codes: (ii) *x*, *y*−1, *z*; (iii) −*x*, *y*+1, −*z*+1/2.
